# SGLT2 Inhibitors and Kidney Protection: Mechanisms Beyond Tubuloglomerular Feedback

**DOI:** 10.34067/KID.0000000000000425

**Published:** 2024-03-25

**Authors:** Ashish Upadhyay

**Affiliations:** Section of Nephrology, Department of Medicine, Boston Medical Center and Boston University Chobanian and Avedisian School of Medicine, Boston, Massachusetts

**Keywords:** CKD, diabetes mellitus, SGLT2

## Abstract

Sodium-glucose cotransporter 2 (SGLT2) inhibitors reduce the risk for kidney failure and are a key component of guideline-directed therapy for CKD. While SGLT2 inhibitors’ ability to activate tubuloglomerular feedback and reduce hyperfiltration-mediated kidney injury is considered to be the central mechanism for kidney protection, recent data from experimental studies raise questions on the primacy of this mechanism. This review examines SGLT2 inhibitors’ role in tubuloglomerular feedback and summarizes emerging evidence on following of SGLT2 inhibitors’ other putative mechanisms for kidney protection: optimization of kidney's energy substrate utilization and delivery, regulation of autophagy and maintenance of cellular homeostasis, attenuation of sympathetic hyperactivity, and improvement in vascular health and microvascular function. It is imperative to examine the effect of SGLT2 inhibition on these different physiologic processes to help our understanding of mechanisms underpinning kidney protection with this important class of drugs.

## Introduction

Sodium-glucose cotransporter 2 (SGLT2) inhibitors block glucose reabsorption in the proximal convoluted tubule of the kidney and increase glycosuria. Canagliflozin, dapagliflozin, and empagliflozin are commonly used SGLT2 inhibitors in clinical practice. While initially developed to lower blood sugar in patients with diabetes mellitus (DM), SGLT2 inhibitors reduce the risk of kidney failure and other major kidney outcomes by 30%–40% over 2–3 years in clinical trials including individuals with CKD, with or without DM.^1–3^ Given these consequential results, SGLT2-inhibitors are now a cornerstone of CKD therapy.

In addition to its role on CKD, SGLT2 inhibitors may also lower the risk of AKI.^4^ AKI risk reduction, however, varies between different classes of SGLT2 inhibitors. Clinical trials show a favorable AKI benefit with empagliflozin and dapagliflozin but not with canagliflozin.^2,5,6^ Similarly, empagliflozin but not canagliflozin reduce histologic markers of tubular damage and biomarkers of AKI in AKI rat models.^7^ The mechanisms underlying these differences may involve variabilities in SGLT2 inhibitors' off-target effects and SGLT2 selectivity.^7^

As clinical trials show a modest blood glucose lowering with SGLT2-inhibitors, their effect on reducing intraglomerular pressure through tubuloglomerular feedback (TGF) activation is speculated to play a central role in kidney protection.^8,9^ SGLT2 inhibitors, however, have pleotropic effects on physiology, and multiple mechanisms likely underpin their clinical benefits. This review examines SGLT2 inhibitors’ role in TGF and summarizes evidence on following of SGLT2 inhibitors’ other putative mechanisms for kidney protection: optimization of kidney's energy substrate utilization and delivery, regulation of autophagy and maintenance of cellular homeostasis, attenuation of sympathetic hyperactivity, and improvement in vascular health and microvascular function.

## TGF and SGLT2 Inhibitors

TGF is an adaptive mechanism that regulates single-nephron GFR (SNGFR) in response to tubular fluid salt concentration at macula densa.^10^ Macula densa releases ATP from the basolateral membrane in proportion to the luminal solute concentration sensed by apical Na^+^-K^+^-2Cl^−^ cotransporters (NKCC2). ATP binds to P2 purinergic receptors on afferent arterioles or cleaves to release adenosine. ATP's action on P2 purinergic receptors and adenosine's action on adenosine A1 receptors result in afferent arteriolar vasoconstriction. In addition, adenosine-mediated effects on juxtaglomerular cells decrease renin secretion and reduce renin-angiotensin-aldosterone (RAAS)–mediated efferent arteriolar vasoconstriction. Increased afferent arterial tone and decreased efferent arterial tone leads to the lowering of intraglomerular pressure and the reduction in SNGFR.

**Figure 1:**
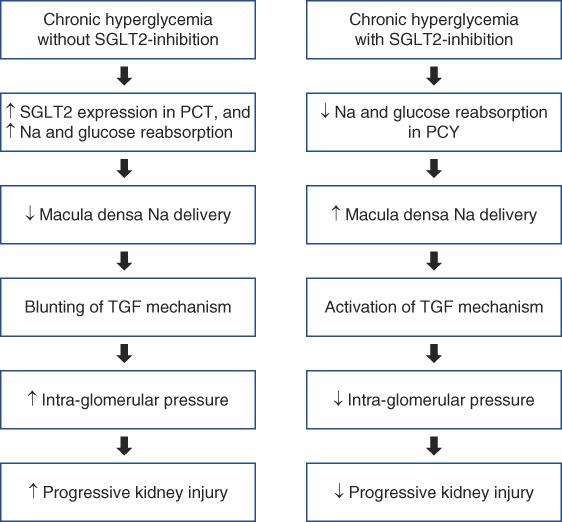
**SGLT2 inhibitors and TGF.** Chronic hyperglycemia increases SGLT2 expression in PCTs leading to enhanced proximal reabsorption of sodium (Na) and glucose and blunting of TGF.^11^ SGLT2 inhibitors block Na and glucose reabsorption in PCT, increase Na and glucose in macula densa, activate TGF, decrease intraglomerular pressure, and reduce hyperfiltration-mediated progressive kidney injury.^8,9^ PCT, proximal convoluted tubule; SGLT2, sodium-glucose cotransporter 2; TGF, tubuloglomerular feedback.

Hyperfiltration, or elevated SNGFR, is an important factor in the initiation and progression of kidney diseases. Hyperfiltration is accentuated in DM because hyperglycemia increases SGLT2 expression leading to enhanced proximal reabsorption and maladaptive inhibition of TGF.^11^ SGLT2 inhibitors increase distal solute delivery, activate TGF, decrease intraglomerular pressure, and reduce hyperfiltration-mediated kidney damage (Figure 1).^8,9^ This results in an average drop in eGFR of 3–6 ml/min per 1.73 m^2^ within few weeks of SGLT2 inhibitor initiation with stabilization and slowing of GFR decline over time.^12^ The magnitude of early GFR drop, however, differs greatly among individuals.^12^ This may be because of the significant variability in the degree of hyperfiltration among patients with DM.^13^ SGLT2 inhibitors reduce GFR when hyperfiltration is present but may not affect GFR in the absence of hyperfiltration.^9^ Therefore, the level of hyperfiltration may determine SGLT2 inhibitors’ ability to modulate TGF.

TGF activation with SGLT2 inhibition is more consistently observed in animal models of diabetic kidney diseases (DKDs) than in models of non-DKDs (Table 1).^14–29^ Similarly, in the dapagliflozin heart failure trial, participants without DM were more likely to be among 30% of participants who did not have a decline in GFR within 14 days of dapagliflozin initiation.^30^ As kidney benefits of SGLT2 inhibitors are similar in individuals with and without DM in clinical trials, mechanisms other than TGF activation must be predominant because TGF activation alone would have more favorably affected patients with DM than those without DM.^1,3^

**Table 1:** Animal studies of SGLT2 inhibitors assessing markers of tubuloglomerular feedback

Author, Study Year	Animal Model	Intervention to Induce SGLT2 Inhibition	Markers Examined to Assess TGF	Summary of Findings Related to TGF	Summary of Findings Related to Changes in Kidney Histology
Vallon *et al.*, 1999^14^	Rat models of STZ-induced diabetes	Phlorizin	SNGFR and distal tubular concentration of electrolytes using micropuncture techniques	Phlorizin reduced SNGFR and increased distal tubular concentration of sodium, chloride, and potassium	N/A
Arakawa *et al.*, 2001^15^	Mice models of obese type 2 diabetes	T-1095	Albuminuria	T-1095 reduced albuminuria	T-1095 suppressed the expansion of glomerular mesangial area
Malatiali *et al.*, 2008^16^	Rat models of STZ-induced diabetes	Phlorizin	GFR, proteinuria	Phlorizin prevented diabetes-associated ↑ in GFR and proteinuria	Phlorizin prevented kidney hypertrophy, but not glomerular hypertrophy
Thompson *et al.*, 2012^17^	Rat models of STZ-induced diabetes	Dapagliflozin	SNGFR and distal tubular chloride concentration using micropuncture techniques	Dapagliflozin lowered SNGFR; SNGFR and distal tubular chloride concentration were inversely correlated	N/A
Vallon *et al.*, 2013^18^	Mice models of STZ-induced diabetes	Gene-targeted SGLT2 knockout	GFR	SGLT2 knockout prevented diabetes-associated ↑ in GFR	N/A
Vallon *et al.*, 2014^19^	Akita mice models of diabetes	Empagliflozin	GFR, albuminuria	Empagliflozin prevented diabetes-induced ↑ in GFR and reduced albuminuria in proportion to hyperglycemia	Empagliflozin attenuated the increase in glomerular size
Tahara *et al.*, 2014^20^	Mice models of STZ-induced diabetes	Ipragliflozin	GFR, albuminuria	Ipragliflozin reduces diabetes-associated ↑ in GFR and albuminuria	N/A
O'Neill *et al.*, 2015^21^	Rat models of STZ-induced diabetes and control rats without diabetes	Phlorizin	GFR, renal plasma flow, renal vascular resistance	Phlorizin reduced GFR in diabetic rats but not in control rats. Phlorizin did not affect renal vascular resistance or renal plasma flow but increased urinary sodium and glucose excretion	N/A
Zhang *et al.*, 2016^22^	Five/six nephrectomy rat models of CKD	Dapagliflozin	GFR, albuminuria	Dapagliflozin did not alter GFR or albuminuria	Dapagliflozin did not attenuate glomerulosclerosis or tubulointerstitial fibrosis
Ansary *et al.*, 2017^23^	Five/six nephrectomy rat models of CKD	Luseogliflozin	Renal blood flow and creatinine clearance	Luseogliflozin did not alter renal blood flow or creatinine clearance but increased urinary sodium and glucose excretion	N/A
Cassis *et al.*, 2018^24^	C57BL/6N mice with unilateral nephrectomy and protein overload proteinuria induced by bovine serum albumin	Dapagliflozin	Albuminuria	Dapagliflozin lowered albuminuria	Dapagliflozin prevented podocyte depletion induced by bovine serum albumin
Kidokoro *et al.*, 2019^25^	Akita mice models of diabetes	Empagliflozin	SNGFR and afferent arteriolar diameter using multiphoton microscope, albuminuria, urinary adenosine excretion	Empagliflozin lowered SNGFR, decreased afferent arteriolar diameter, and increased urinary adenosine concentration. Adenosine A1 receptor antagonist abolished the effects of empagliflozin	N/A
Thomson and Vallon, 2021^26^	Rat models of STZ-induced diabetes	Ertugliflozin	GFR, glomerular capillary pressure, and proximal reabsorption using micropuncture techniques	Ertugliflozin reduced GFR, glomerular capillary pressure, and proximal reabsorption tubular reabsorption	N/A
Tauber *et al.*, 2021^27^	Unilateral nephrectomy mice models of CKD on normal and high salt diet	Empagliflozin	GFR	Empagliflozin reduced GFR in mice models of normal salt diet but not in mice models of high salt diet	Empagliflozin did not affect renal fibrosis in mice models of high salt diet
Zeng *et al.*, 2022^28^	Five/six nephrectomy rat models of CKD on a high salt diet	Empagliflozin	Urinary adenosine excretion	Empagliflozin did not affect urinary adenosine excretion	Empagliflozin reduced renal fibrosis
Chen *et al.*, 2023^29^	Five/six nephrectomy rat models of CKD	Empagliflozin	Creatinine clearance, albuminuria, urinary adenosine excretion	Empagliflozin decreased albuminuria and increased urinary adenosine concentration. Empagliflozin, however, increased creatinine clearance	Empagliflozin improved renal interstitial fibrosis and glomerulosclerosis

N/A, not available; STZ, streptozocin; SNGFR, single-nephron GFR; TGF, tubuloglomerular feedback.

The relationship between SGLT2 inhibition and TGF may be different in type 1 and type 2 DM. In individuals with type 1 DM, empagliflozin significantly lowers hyperfiltration and increases renovascular resistance, as expected with the activation of TGF.^9^ On the contrary, in individuals with type 2 DM, dapagliflozin reduces hyperfiltration without increasing renovascular resistance, suggesting the role of efferent arteriolar vasodilation rather than afferent arteriolar vasoconstriction.^31^ The differences in the severity of preglomerular vascular disease between individuals with type 1 and type 2 DM may have led to this variant observation.^31^

**Figure 2:**
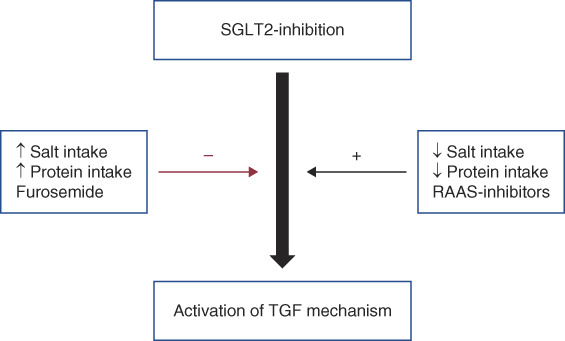
**Salt intake, protein intake, and medication SGLT2 inhibitor's effect on TGF.** SGLT2 inhibitors may activate TGF mechanism.^8,9^ High salt intake, high protein intake, and furosemide may blunt SGLT2 inhibitor's effects on TGF.^27–29,32,33^ Low salt intake, low protein intake, and RAAS inhibitors may potentiate SGLT2 inhibitor's effects on TGF. RAAS, renin-angiotensin-aldosterone.

Salt intake may influence SGLT2 inhibitors' role on TGF (Figure 2). SGLT2 inhibitors reduce GFR, lower proteinuria, and increase urinary adenosine excretion (a marker of TGF activation) in rodents on a normal salt diet, but not on a high salt diet.^27,29^ Interestingly, empagliflozin does not affect urinary adenosine excretion but still significantly lowers kidney fibrosis in experiments involving high salt diet rat models of CKD.^28^ These observations suggest that a high salt diet blunts TGF activation, and benefits of SGLT2 inhibition in a setting of high salt diet likely involve other mechanisms.

The activation of TGF is affected by macula densa's responsiveness to tubular solute concentration. For example, high protein diet can blunt TGF activation by increasing the expression and activity of nitric oxide synthase in macula densa.^32^ Similarly, as NKCC2 mediates TGF sensing, blocking of NKCC2 by furosemide also blunts SGLT2 inhibition's effect on TGF.^33^ Data from clinical trials, however, show that neither dietary protein intake nor the use of diuretics significantly alter benefits observed with SGLT2-inhibitors.^1,34^ Concomitant use of RAAS blockers, agents that potentiate TGF, may have helped to balance the potential effect of high protein diet and loop diuretics on TGF.

Some TGF adaptation may occur over time with SGLT2 inhibition. In animal models, dapagliflozin, as expected, acutely activates TGF, but there is a partial relaxation of TGF tone after 10–12 days of dapagliflozin exposure.^17^ Luminal solute concentration at the macula densa that drive TGF activation may diminish over time because SGLT2 inhibition's blocking of proximal reabsorption is accompanied by a compensatory rise in SGLT1-mediated glucose reabsorption and vasopressin, aldosterone, and uromodulin-mediated solute and water reabsorption in the downstream nephron segments.^35,36^ In addition, SGLT1's sensing of SGLT2 inhibition–mediated increase in luminal glucose at the macula densa promotes the activity and expression of nitric oxide synthase, which improves nitric oxide availability and blunts the TGF-induced afferent vasoconstriction.^37^

While the influence of SGLT2 inhibitors on TGF is likely important in selected settings, it is essential to explore other potential mechanisms for kidney protection with SGLT2 inhibitors given the magnitude of benefits observed across broad patient subgroups.

## Optimization of Kidney's Energy Substrate Utilization and Delivery

While >90% of glucose reabsorption in the kidney occurs through SGLT2, pharmacologic SGLT2 blockade only inhibits 30%–50% of glucose reabsorption because of the compensatory rise in distal glucose reabsorption.^36^ Therefore, SGLT2 inhibitors result in 50–90 g of glucose or 200–360 kcal of energy loss per day in individuals filtering approximately 180 g of glucose daily. Energy loss is higher with higher GFR and blood glucose concentration. This energy loss leads to a state akin to calorie restriction and triggers physiologic processes that promote the efficient utilization of energy substrates.

**Figure 3:**
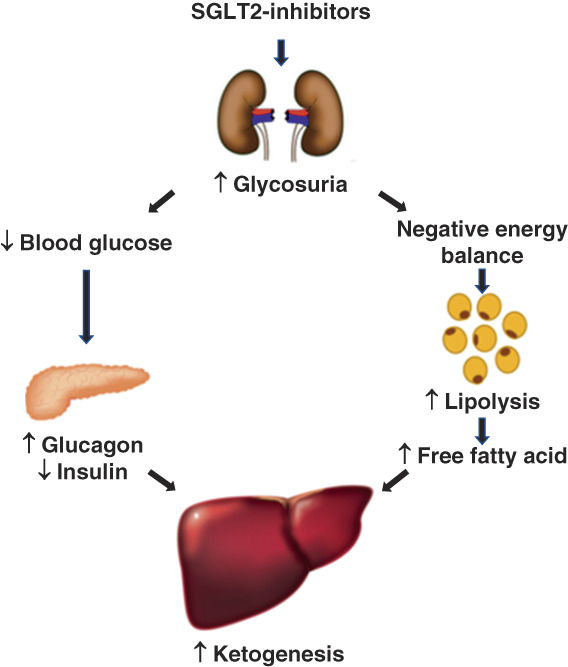
**SGLT2 inhibitors and ketogenesis.** SGLT2 inhibitors increase glycosuria, lower blood glucose, and create a negative energy balance.^38^ These mechanisms stimulate hepatic ketogenesis by promoting lipolysis, lowering pancreatic insulin secretion, and increasing pancreatic glucagon secretion.^38,39^

Ferranni and colleagues first proposed a thrifty substrate hypothesis in 2016 to explain SGLT2 inhibitors’ observed cardiorenal benefits.^38^ The central rationale for this hypothesis is that SGLT2 inhibitors promote lipolysis by inducing negative energy balance and stimulate ketogenesis by increasing hepatic delivery of free fatty acids and lowering insulin-to-glucagon ratio (Figure 3).^38^ Ketones then serve as an efficient energy source for organs under stress. An increase in plasma concentration of *β*-hydroxybutyrate, a ketone body, is observed with SGLT2-inhibition,^39^ and kidneys and heart have a large capacity for using *β*-hydroxybutyrate as an energy source.^40^

Organs under stress take up *β*-hydroxybutyrate through an insulin-independent transport mechanism.^38^ Inside cells, mitochondrial *β*-hydroxybutyrate dehydrogenase converts *β*-hydroxybutyrate to acetyl coenzyme A through multiple catalytic steps.^38^ Acetyl coenzyme A enters into the tricarboxylic acid cycle for oxidative phosphorylation leading to the generation of ATP.^38^ Compared with pyruvate, the product of glucose metabolism, *β*-hydroxybutyrate oxidation requires less oxygen and provides better mitochondrial efficiency.^38^ In addition, *β*-hydroxybutyrate serves as a signaling molecule that activates adaptive stress response pathways, suppresses oxidative stress, and lowers inflammation.^41,42^ Therefore, in a state of kidney stress, *β*-hydroxybutyrate may play a role in mitigating kidney injury.

**Figure 4:**
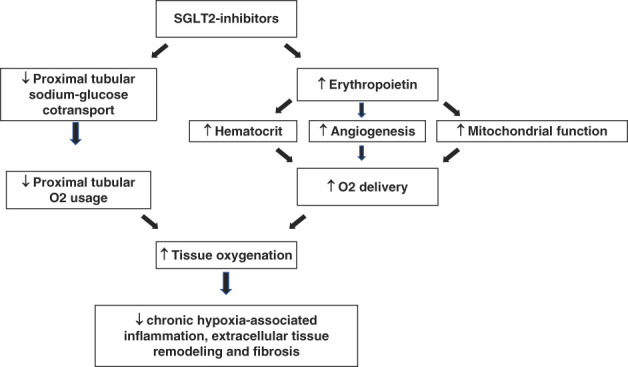
**SGLT 2 inhibitors and tissue oxygenation.** SGLT2 inhibitors improve tissue oxygenation in kidneys by decreasing proximal tubular oxygen (O_2_) usage.^43^ SGLT2 inhibitors potentially increase O_2_ delivery through erythropoietin-mediated increase in hematocrit, angiogenesis, and mitochondrial function. Improvement in tissue oxygenation lowers hypoxia-associated kidney damage.^44–47^

In addition to improving energy substrate utilization through ketogenesis, SGLT2 inhibitors may also increase energy substrate delivery, lower oxygen consumption, and reduce hypoxia-induced kidney damage (Figure 4). In clinical trials, SGLT2 inhibitors increase hematocrit by 2%–4%, likely by stimulating erythropoietin secretion.^44^ Higher hematocrit may improve tissue oxygen delivery, and higher erythropoietin may improve mitochondrial function, promote angiogenesis, and reduce inflammation.^45–47^ A mediation analysis of a large clinical trial showed that changes in hematocrit mediated >50% of the favorable effect of empagliflozin on cardiovascular mortality.^48^ Interestingly, however, improvement in renal cortical oxygenation occurred without an increase in cortical perfusion or oxygen delivery in a trial involving participants with type 1 DM.^43^ This observation suggests that SGLT2 inhibition may be improving kidney oxygenation in this trial by blocking high oxygen-consuming proximal solute reabsorption rather than by increasing oxygen supply. Because chronic hypoxia promotes inflammation and fibrosis, SGLT2 inhibitors may be providing kidney protection by ameliorating deleterious effects of chronic hypoxia.

SGLT2 inhibitors may also improve metabolism and energy utilization through its effect on leptin-adiponectin ratio. Leptin is an adipokine that is associated with insulin resistance, inflammation, and oxidative stress.^49^ Adiponectin is an anti-inflammatory adipokine that enhances insulin sensitivity.^49^ A high leptin-adiponectin ratio is associated with CKD,^49^ and adiponectin reduces CKD risk.^50^ SGLT2-inhibitors significantly decrease leptin and increase adiponectin levels in clinical trials involving patients with type 2 diabetes.^51^ Mechanisms underlying these findings have not been fully elucidated.

## Regulation of Autophagy and Maintenance of Cellular Homeostasis

**Figure 5:**
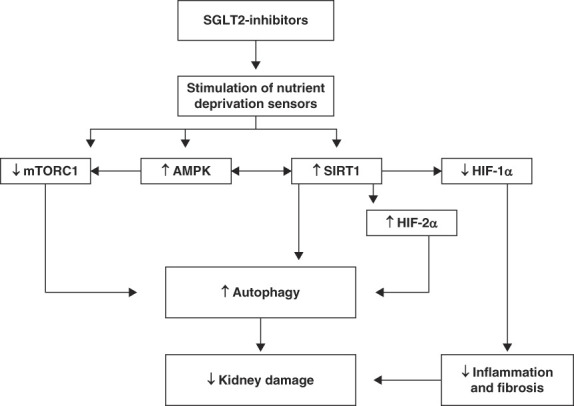
**SGLT 2 inhibitors and autophagy.** SGLT2 inhibitors create negative energy balance which stimulates nutrient deprivation sensors in tissues.^53^ Tissues respond to nutrient deficiency by inducing pathways that inhibit mTORC1 and stimulate AMPK and SIRT1.^54–56^ SIRT1 stimulates AMPK and HIF-2*α* and suppresses HIF-1*α*.^53,57,58^ These pathways ultimately contribute to the reduction in kidney damage by improving autophagy and lowering inflammation and fibrosis. AMPK, 5′adenosine monophosphate-activated protein kinase; HIF, hypoxia-inducible factor; mTORC1, mammalian target of rapamycin protein complex 1; SIRT1, sirtuin 1.

Autophagy is a lysosome-dependent pathway for the degradation and recycling of cytosolic components that provides new building blocks necessary for tissue repair. Autophagy deficiency contributes to kidney damage.^52^ In recent years, there is an emerging understanding that SGLT2 inhibitors activate autophagy by favorably influencing mammalian target of rapamycin (mTOR), 5′adenosine monophosphate-activated protein kinase (AMPK), sirtuin 1 (SIRT1), and hypoxia-inducible factor (HIF) signaling pathways (Figure 5).^54–56^

mTOR protein complex 1 (mTORC1) is a serine-threonine kinase protein complex that integrates nutrient signals, including from glucose and amino acid, to stimulate anabolism and suppress autophagy in a state of calorie excess.^59^ Proximal tubular mTORC1 is believed to play a significant role in DKD.^60^ SGLT2 inhibitors reduce the activity of mTORC1 in proximal tubular cells and prevents tubulointerstitial fibrosis.^60^ AMPK, unlike mTOR, is a sensor of low intracellular energy (low ATP to 5′adenosine monophosphate ratio) that stimulates catabolism and activates autophagy by inhibiting mTORC1 in a state of calorie deficit.^61^ The catabolic process stimulated by AMPK then supplies ATP to energy-deficient cells. Canagliflozin, likely by augmenting calorie loss, activates AMPK-mediated autophagic stimulation independent of insulin or glucagon signaling.^62^ Multiple studies demonstrate that SGLT2 inhibitors stimulate autophagy through mTOR-AMPK–mediated mechanisms and contribute to kidney protection in a wide variety of kidney insults.^63–65^

SIRT1 is a nicotinamide adenine dinucleotide–dependent deacetylase that acts as a nutrient deprivation sensor.^66^ SIRT1 deacetylates tumor protein 53, which accelerate autophagy activating pathways.^67^ During glucose deprivation, SIRT1 and AMPK stimulate each other to augment autophagy and mitochondrial biogenesis.^57^ SIRT1 also suppresses mTORC1 independent of AMPK.^68^ SIRT1 deficiency accelerates kidney damage, and SIRT1 activation alleviates kidney injury.^69,70^ Experimental studies show that SIRT1 expression increases in animal models exposed to SGLT2 inhibitors.^71,72^

HIFs are a family of transcription factors that regulate cellular responses to hypoxia. HIF-1*α* and HIF-2*α* are isoforms of HIF that are upregulated by hypoxia and promote gene expressions that enhance oxygen delivery and reduce oxygen consumption.^53^ Specifically, HIF-1*α* activation increases inflammation, fibrosis, angiogenesis, and autophagic clearance of mitochondria whereas HIF-2*α* activation decreases inflammation and fibrosis and increases erythropoietin production and autophagic clearance of peroxisomes.^53^ In diseases of over nutrition, such as DM and obesity, HIF-1*α* is overactive, and HIF-2*α* is suppressed in kidney tissues promoting inflammation and fibrosis.^53,73^ SGLT2 inhibition suppresses hypoxia-induced HIF-1*α* expression in human proximal tubules and reduces tubular injury and interstitial fibrosis.^74^ In addition, SGLT2 inhibitors may also increase HIF-2*α* expression through SIRT1-dependent mechanism.^58^ Thus, SGLT2 inhibitors may be providing kidney protection by restoring the balance between HIF-1*α* and HIF-2*α* expressions in kidney cells.

SGLT inhibitors may have a role in maintaining podocyte homeostasis. Albumin overload increases SGLT2 expression on podocyte surface and leads to the remodeling of F-actin and *α*-actinin-4 filaments and loss of *β*1-integrins, proteins that are vital for podocyte integrity and function.^24^ SGLT2 inhibition directly ameliorates albumin overload–induced cytoskeletal rearrangement and decreases podocyte dysfunction and loss.^24^ SGLT2 inhibition may also alleviate podocyte injury and loss by dampening inflammation and activating autophagy through the inhibition of mTORC1 signaling.^75^

SGLT2 inhibition may favorably influence the role of macrophages in kidney injury and repair. During acute injury, chemokines and complement components released by damaged cells promote monocyte recruitment and monocyte's differentiation into proinflammatory M1 macrophages.^76^ During chronic repair phase, monocytes primarily differentiate into anti-inflammatory M2 macrophages and M1 macrophages polarize to M2 macrophages.^76^ While M2 macrophages are anti-inflammatory, their polarization may also result in profibrotic M2 subtypes.^76^ SGLT2 inhibitors impede macrophage infiltration in kidney tissues, promote M1–M2 polarization, and inhibit the polarization of M2 macrophages into profibrotic subtypes.^77,78^

SGLT2 inhibitors may help maintain cellular redox homeostasis and reduce oxidative stress in kidney tissues by activating the Kelch-like ECH-associated protein 1–nuclear factor erythroid 2-related factor 2 (Keap1-Nrf2) pathway.^79^ Keap1-Nrf2 activation is postulated to reduce arteriolar tone and improve nitric oxide bioavailability.^79^ Consistent with these hypotheses, Keap1-Nrf2 activation increases GFR and renal blood flow in animal studies,^79,80^ an effect that is opposite to what is expected through TGF activation. While Keap1-Nrf2 activation may increase GFR without affecting intraglomerular pressure,^80^ more mechanistic studies are needed to better understand how SGLT2 inhibitor's effect on Keap1-Nrf2 system modulates its effect on glomerular hemodynamics through TGF.

## Attenuation of Sympathetic Hyperactivity

**Figure 6:**
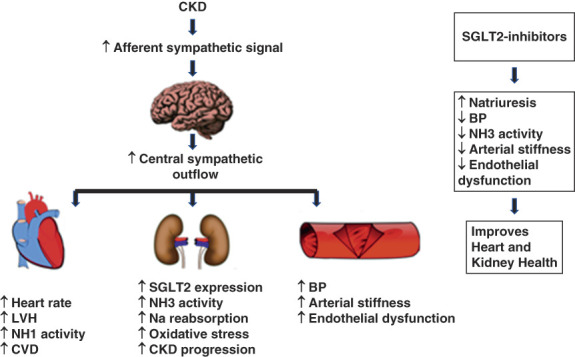
**CKD, SGLT2 inhibitors, and sympathetic tone.** There is an increase in sympatho-excitatory afferent signals from damaged kidneys to the brain in CKD.^81,82^ This leads to an increase in central sympathetic outflow. Increased sympathetic outflow increases heart rate and BP, contributes to LVH and CVDs, increases SGLT2 expression in the PCT, activates NH1 and NH3, increases sodium retention, increases oxidative stress, contributes to arterial stiffness, and worsens endothelial function.^81–89^ SGLT2 inhibitors mitigate these adverse effects of increased sympathetic tone by promoting natriuresis, lowering BP, inhibiting NH3 activity, improving arterial function, and improving endothelial function.^87,90–96^ CVD, cardiovascular disease; LVH, left ventricular hypertrophy; NH1, Na^+^/H^+^ exchanger isoform 1; NH3, Na^+^/H^+^ exchanger isoform 3.

Hyperactivity of sympathetic nervous system (SNS) is associated with CKD, contributes to CKD progression, and is a marker of poor prognosis (Figure 6).^83^ Abnormal RAAS activation, sympatho-excitatory afferent signals from damaged kidneys, inhibition of nitric oxide's sympatho-inhibitory and vagotonic effects, and increase in oxidative stress are some of the mechanisms for increased sympathetic tone in CKD.^81,82^ SNS stimulation promotes proximal tubular sodium reabsorption, upregulates SGLT2 expression, and increases translocation of SGLT2 to cell membrane.^84,85^ SNS stimulation also activates luminal Na^+^/H^+^ exchanger isoform 3 (NH3) in proximal tubules.^86^ SGLT2 and NH3 are linked, and the natriuretic effect of SGLT2-inhibitors is partially through its inhibition of NH3-mediated sodium reabsorption.^87^ SGLT2 inhibitors, therefore, mitigate sodium retention that accompanies SNS activation.

SGL2 inhibitors may also attenuate cardiac risks from sympathetic hyperactivity by directly conferring favorable ion homeostasis in cardiomyocytes. SGLT2 inhibitors inhibit Na^+^/H^+^ exchanger 1, late sodium channel current during action potential, and calcium/calmodulin-dependent protein kinase-II, leading to a reduction in sodium and calcium overload in cardiomyocytes.^88,89^ Lower intracellular sodium and calcium enhances mitochondrial function, decreases the risks for arrhythmias, and improves systolic and diastolic function.

SGLT2 inhibitors lower systolic BP without increasing heart rate in clinical trials, contrary to what is expected for drugs that cause natriuresis.^97^ This observation led to the hypothesis that SGLT2 inhibitors, in addition to causing natriuresis, may be alleviating aberrant SNS stimulation, because low heart rate is associated with low sympathetic tone.^98^ Consistent with this hypothesis, SGLT2 inhibitors lower the markers of SNS activity like norepinephrine, neuropeptide Y and tyrosine hydroxylase levels, norepinephrine turnover, arterial pressure lability, and salt sensitivity in animal studies.^90–92^ Similarly, limited data also suggest the favorable effect of SGLT2 inhibition on SNS activity in human participants.^93^ These observations indicate that SGLT2 inhibitors may partly be contributing to heart-kidney protection through the attenuation of sympathetic hyperactivity seen in CKD.

## Improvement in Vascular Health and Microvascular Function

Arterial stiffness and endothelial dysfunction, important mechanisms for vascular diseases, correlate with kidney dysfunction and albuminuria.^99–101^ SGLT2 inhibitors have been shown in clinical trials to modestly lower pulse wave velocity, a measure of arterial stiffness, and improve brachial artery flow mediated dilation, a measure of endothelial dysfunction.^94–96^ In addition, there may be a connection between SGLT2 inhibitors and endothelin-1, a potent vasoconstrictor. Post hoc analyses of clinical trials suggest that SGLT2 inhibitors may lower endothelin-1 level and mitigate fluid retention seen with endothelin receptor antagonists.^102,103^

Favorable effect on vascular function noted in SGLT2 inhibitor clinical studies is consistent with data from basic science research. Mice studies show that SGLT2 inhibitors improve endothelial function and reduce molecular signals that aggravate arterial stiffness.^104^ SGLT2 inhibitors may improve endothelial function and promote vasorelaxation by inhibiting the secretion of endothelial proinflammatory cytokines, reducing mitochondrial and cytoplasmic reactive oxygen species, and increasing endothelium-derived nitric oxide level.^105–107^ SGLT2 inhibitors may also induce vasodilation directly by activating protein kinase G, voltage-gated potassium channels, and the intrarenal angiotensin-(1–7)/angiotensin-converting enzyme 2/Mas axis.^108–110^ The activation of angiotensin-(1–7)/angiotensin-converting enzyme 2/Mas axis results in vasodilation through the release of bradykinin, prostaglandin, and endothelin-dependent nitric oxide.^109,110^

SGLT2 inhibitors may also improve vascular function by mobilizing bone marrow-derived CD34-positive circulating progenitor cells and restoring the vascular repair system.^111^ Using a series of experiments in samples from participants with DM and recent myocardial infarction, Hess and colleagues showed that empagliflozin increases circulating provascular progenitor cells and reduces systemic oxidative stress creating a microenvironment that is more permissive to blood vessel regeneration.^111^ Additional studies are needed to confirm this intriguing hypothesis.

## Conclusion

SGLT2 inhibitors reduce the risk for kidney failure and are now a cornerstone of CKD therapy. In addition to activating TGF, lowering intraglomerular pressure and lowering hyperfiltration-mediated kidney damage, SGLT2 inhibitors promote multiple other favorable physiologic pathways that contribute to kidney health. Optimization of energy substrate utilization and delivery, promotion of cellular renewal through the activation of autophagic pathways, attenuation of sympathetic tone, and improvement in vascular function are some of the potential mechanisms for SGLT2 inhibitors’ observed benefits in CKD. Future research is needed to understand whether these favorable physiologic changes are sustained with chronic treatment and whether SGLT2 inhibitors have a role in preventing CKD in at-risk individuals.
